# Chronic lymphocytic leukaemia/small lymphocytic lymphoma and mantle cell lymphoma: from early lesions to transformation

**DOI:** 10.1007/s00428-022-03460-y

**Published:** 2022-12-01

**Authors:** Birgitta Sander, Elias Campo, Eric D. Hsi

**Affiliations:** 1 Department of Laboratory Medicine, Division of Pathology, Karolinska Institutet and Karolinska University Hospital, Stockholm, Sweden; 2 Laboratory of Pathology Hospital Clinic of Barcelona, University of Barcelona, Barcelona, Spain; 3 Institute of Biomedical Research August Pi I Sunyer (IDIBAPS), Barcelona, Spain; 4 Department of Pathology, Wake Forest University School of Medicine, Winston-Salem, NC USA

**Keywords:** Chronic lymphocytic leukaemia, B-cell prolymphocytic leukaemia, Mantle cell lymphoma, Classification

## Abstract

The International Clinical Advisory Committee reviewed advances in our understanding of the clinicopathologic and biologic features of chronic lymphocytic leukaemia/small lymphocytic lymphoma, B-cell prolymphocytic leukaemia, and mantle cell lymphoma since the revised 4th edition of the WHO Classification of Tumours of the Haematopoietic and Lymphoid Tissues. Discussions amongst pathologists, clinicians, and molecular geneticists around these diseases focussed on incorporating new knowledge into the next classification system. In this manuscript, we review these disease entities and incorporate results of these deliberations, including advances in our understanding of early lesions and transformation.

## Introduction

Chronic lymphocytic leukaemia/small lymphocytic lymphoma (CLL/SLL) and mantle cell lymphoma (MCL) are two lymphoid neoplasms characterized by the clonal expansion of mature small CD5^+^ B cells that may involve the bone marrow, blood, lymphoid tissues, and extranodal sites [1]. Tumour cells in both diseases differ in the morphological, phenotypic, and genomic profiles. The clinical evolution of patients with either CLL/SLL or MCL may be very heterogeneous, and one major source of this heterogeneity is related to the cell of origin of the tumour. Both CLL/SLL and MCL include two major clinical and molecular subtypes related to their origin in naïve-like or memory-like cells. The subtype derived from naïve-like cells carry no or low load of somatic mutations in the immunoglobulin heavy- and light-chain variable (IGHV) regions and have more unfavourable evolution in both diseases. In contrast, the subtype derived from memory-like cells carry a higher load of IGHV somatic mutations and have a more indolent course. Despite this different cell of origin, both subgroups of CLL/SLL and MCL have restricted immunoglobulin gene repertoire suggesting dependency on B cell receptor signalling/antigen drive for their development [1]. Early lesions are recognized in both entities as monoclonal B-cell lymphocytosis (MBL) in CLL and in situ mantle cell neoplasia. Transformation to very aggressive forms occurs in both molecular subtypes of CLL/SLL and MCL adopting different histological appearances with poor prognosis. Studies in the last years have provided new information that refines the understanding of these entities and has practical implications that have been included in the 2022 International Consensus Classification (ICC) [2].

### Monoclonal B-cell lymphocytosis and chronic lymphocytic leukaemia

#### Monoclonal B-cell lymphocytosis (MBL)

MBL is the clonal proliferation of lymphocytes in the peripheral blood with the absolute clonal cell count defined as less than 5.0 × 10^9^/L. The prevalence depends on the patient population studied, and the sensitivity of the flow cytometry assay used. With standard sensitivity and clinical laboratory assays, approximately 3.5% of individuals over 40 years of age have an MBL [3, 4]. The great majority of cases have the immunophenotype of CLL, and the relative risk for progression to CLL requiring treatment is approximately 1%/year [5]. Besides CLL-type MBL, atypical CLL (CD5 + /bright CD20 + /variable CD23 + /bright surface immunoglobulin) and non-CLL (CD5-dim/negative without other phenotypic markers indicative a specific lymphoproliferative disorder) types can be seen, the latter perhaps related to splenic marginal zone lymphoma. CLL-type MBL is divided into low count (< 0.5 × 10^9^/L) or high count (≥ 0.5 × 10^9^/L), where low count cases have negligible risk for progression to CLL [6].

High-risk molecular abnormalities seen in CLL are under-represented in MBL. Immunogenetic analysis of low-count MBL shows that IGHV rearrangements common in CLL are under-represented or not used. Mutated IGHV genes are more frequent in low-count MBL compared to high-count MBL, while the use of stereotyped receptors is rare in low-count MBL [7]. Whole genome sequencing in a limited number of cases of low-count MBL, high-count MBL, and stable Rai-stage 0 CLL cases shows low genomic complexity, few exonic mutations in known CLL driver genes, and little that distinguishes them [8].

The concept of a tissue form of MBL has been raised [7, 9, 10]. Lymph nodes or other extranodal tissues, usually biopsied for reasons other than suspicion for lymphoma, may incidentally be found to contain low levels of monoclonal B-cells by flow cytometry [7, 10]. Studies have suggested a size cut-off of 1.5 cm, non-distorted architecture, and lack of proliferation centres as features to segregate partial involvement by SLL from a tissue-based MBL. Careful immunohistochemical analysis can highlight the abnormal cells in some, but not all cases. Such cases may not progress, and evaluation of the peripheral blood will, in most cases, demonstrate a high count MBL [9, 10].

## Chronic lymphocytic leukaemia/small lymphocytic lymphoma (CLL/SLL)

### Definition and clinical features

CLL/SLL is the most common low-grade B-cell leukaemia in the Western world with an incidence of 4.9 per 100,000 people/year and median age at diagnosis of 70 years. Women are more frequently affected (M: F = 0.53). While the epidemiology of CLL/SLL is similar between the USA and Europe, the incidence appears lower in Asian countries (https://seer.cancer.gov/statfacts/html/clyl.html; 2021, [11]).

The definition of CLL relies on the absolute count of ≥ 5 × 10^9^ leukemic cells/L in the blood. Cases with nodal, splenic, or extramedullary involvement but less than this degree of lymphocytosis are termed SLL. The malignant cell type is the same. Virtually all cases of CLL are preceded by MBL [5]. Many patients with CLL/SLL are asymptomatic and are diagnosed incidentally during investigation for an unexplained leukocytosis. Symptomatic CLL/SLL patients may present with lymphadenopathy, organomegaly, anaemia, thrombocytopenia, or, less commonly, autoimmune manifestations, paraproteinemia (usually IgM), or recurrent infection [11].

### Pathologic and immunophenotypic features

The peripheral blood smear shows a lymphocytosis composed of small mature lymphocytes with scant cytoplasm and condensed chromatin that can impart a cracked or “soccer ball” appearance. Slight nuclear irregularity (often associated with trisomy 12) or more abundant cytoplasm may sometimes be seen. Prolymphocytes are intermediate-sized (1.5 times the size of a lymphocyte) with open/dispersed chromatin and a visible central nucleolus. They are usually < 15% of lymphocytes but may be higher. Prolymphocytes are always below 55% of lymphocytes. Cases with > 55% prolymphocytes meet the criteria for B-cell prolymphocytic leukaemia (B-PLL) [12]. Flow cytometry is mandatory, and the typical phenotype is CD19 + , CD20 + (dim), CD5 + , CD10 − , CD23 + , CD43 + , CD79b (dim), CD81(dim), ROR1 + , CD200 + , with monotypic surface immunoglobulin light chain (dim or less commonly undetectable). Core diagnostic markers consist of CD19, CD5, CD20, CD23, Kappa, and Lambda. Additional markers potentially useful for differential diagnosis include CD43, CD79b, CD81, CD200, CD10, and ROR1 [13]. Expression of CD38 or CD49d has been associated with adverse prognosis.

In tissue, SLL/CLL manifests as a diffuse proliferation of small round lymphocytes with mature chromatin that totally distorts or effaces the lymph node architecture. Prolymphocytes and paraimmunoblasts are admixed and variable in number. They may be present in clusters to form proliferation centres. Mitoses, when present, are generally found associated with proliferation centres. Uncommon cases with increased proliferation centres that are highly proliferative (> 40% Ki67 index) or that become confluent to fill a 20 × microscopic objective field have a more aggressive course and have been termed accelerated CLL/SLL [14]. Immunohistochemistry shows expression of CD5, CD19, CD20, CD23 while cyclin D1, SOX11 and CD10 are negative. Of note, focal cyclin D1 expression can occur in proliferation centres in up to 20% of cases of CLL/SLL without the presence of the t(11;14) or SOX11 expression [15]. Expression of LEF1 is seen in the great majority of CLL/SLL cases whereas it is absent in the great majority of MCL and marginal zone lymphoma (including CD5 + cases) [16].

### Genetic features

Fluorescence in situ hybridization (FISH) studies for deletions of 13q14, 11q13, and 17p13, and trisomy 12 stratify patients into risk groups [11] (Table 1). G-banding also can provide prognostic information independent of other factors by assessing overall genomic complexity. Definitions are in flux and while cases with ≥ 3 abnormalities have an adverse prognosis, a recent study suggests ≥ 5 abnormalities recognize a particularly high-risk group of patients [17]. Application of high-resolution genome-wide DNA microarrays has also shown cases with ≥ 5 copy number abnormalities to identify patients with adverse outcomes in terms of time to first treatment and overall survival, independent of other factors [18]. While promising, DNA microarrays are not routinely applied, and further technical and interpretive standardization and validation are needed.

**Table 1:** Median survival of CLL patients by structural abnormality. Adapted from [22]

Abnormality	Median survival (months)
13q deletion	133
Normal karyotype	111
Trisomy 12q	114
11q deletion	79
17p deletion	32

#### IGHV mutational status and stereotype

Somatic hypermutation introduces diversity into the immunoglobulin receptor genes of B-cells. CLL cases with ≥ 98% similarity of the IGHV sequence (unmutated CLL, U-CLL) have a poor outcome compared to those with < 98% homology (mutated CLL, M-CLL) [11]. Numerous studies have validated this finding as an independent prognostic factor for clinical endpoints such as survival and time to first treatment. IGHV mutational status has been incorporated into clinical guidelines and scoring systems, such as the international CLL international prognostic score, for the management of patients and is considered a requirement in general practise and clinical trials (Table 2) [19].

**Table 2:** CLL international prognostic index validation dataset. Adapted from [19]

	CLL IPI score	Patients (%)	Median OS (95% CI)	5 year OS (95% CI)	10 year OS (95% CI)
Low	0–1	186 (32%)	NR	90.7% (86.4–95.1)	86.5% (80.5–92.4)
Intermediate	2–3	200 (34%)	104 (84–123)	79.8% (83.9–85.8)	40.1% (29.3–50.9)
High	4–6	147 (25%)	63 (51–73)	52.8% (44.5–61.1)	16.1% (6.7–25.4)
Very high	7–10	52 (9%)	31 (20–39)	18.6% (7.5–29.7)	0% (NE)

*NR* not reached, *NE* not evaluable

Detailed immunogenetic analysis has resulted in the concept of stereotypy of the B-cell receptor (BCR), in which similarities in the amino acid patterns of the BCR are analysed and cases grouped based on sequence of the IGH V, D, and J genes. Large-scale sequencing has revealed 29 major stereotypes with an individual frequency > 0.2%, accounting for 41% of all CLL cases. Subset #8 is associated with increased risk for Richter transformation [20]. Subset #2 includes cases using the IGHV3-21/IGLV3-21 genes and has been associated with poor prognosis, regardless of the SHM mutation status [20]. Recently, a particular mutation, IGLV3-21^R110^, that induces autonomous IG signalling, has been detected in all stereotype #2 cases, but also in CLL without this BCR configuration. It is present in 5–18% of CLL and confers poor prognosis independent of the IGHV mutational status [21]. While not yet routinely assessed in clinical practice, BCR stereotype and IGLV3-21^R110^ may inform risk stratification and therapy choice in select cases. In the future, it may become mandatory for these purposes.

#### Mutational profile

CLL with *TP53* abnormalities, either deletion or mutation, have poor outcomes compared to wild-type cases and do not respond well to conventional chemoimmunotherapy [22]. *TP53* deletions and mutations have similar implications, and assessment for these alterations is now required to guide therapy [23, 24]. Technical variability exists with traditional Sanger sequencing and next generation sequencing (NGS) strategies can be employed. *TP53* exons 4–10 are often tested due to a higher frequency of mutations but other coding areas may harbour deleterious mutations and sequencing of the entire coding region may be desirable. The detection of low-level *TP53* mutations (5–10% variant allele fraction) has been consistently associated with poor outcome [25, 26]. However, introduction in the clinical practice of this low-level detection requires standardization and confirmation of the results in clinical trials [27].

Large-scale genomic studies have defined the mutation landscape of CLL. Among the most commonly (≥ 3%) mutated genes are *SF3B1*, *TP53*, *NOTCH1*, *MYD88*, *ATM*, *XPO1* and *CHD2 *[28, 29]. Besides *TP53*, other genes such as *SF3B1*, *NOTCH1*, *ATM*, and *BIRC3* are emerging as genes of interest for prognosis, which may be related to their clonal or subclonal distribution [25, 30]. Genomic profiling using NGS gene panels is a promising tool for risk stratification and possibly therapy selection in CLL that could integrate the detection of IG mutational status, gene mutations, and DNA copy number alterations.

### Prognosis

**Fig. 1:**
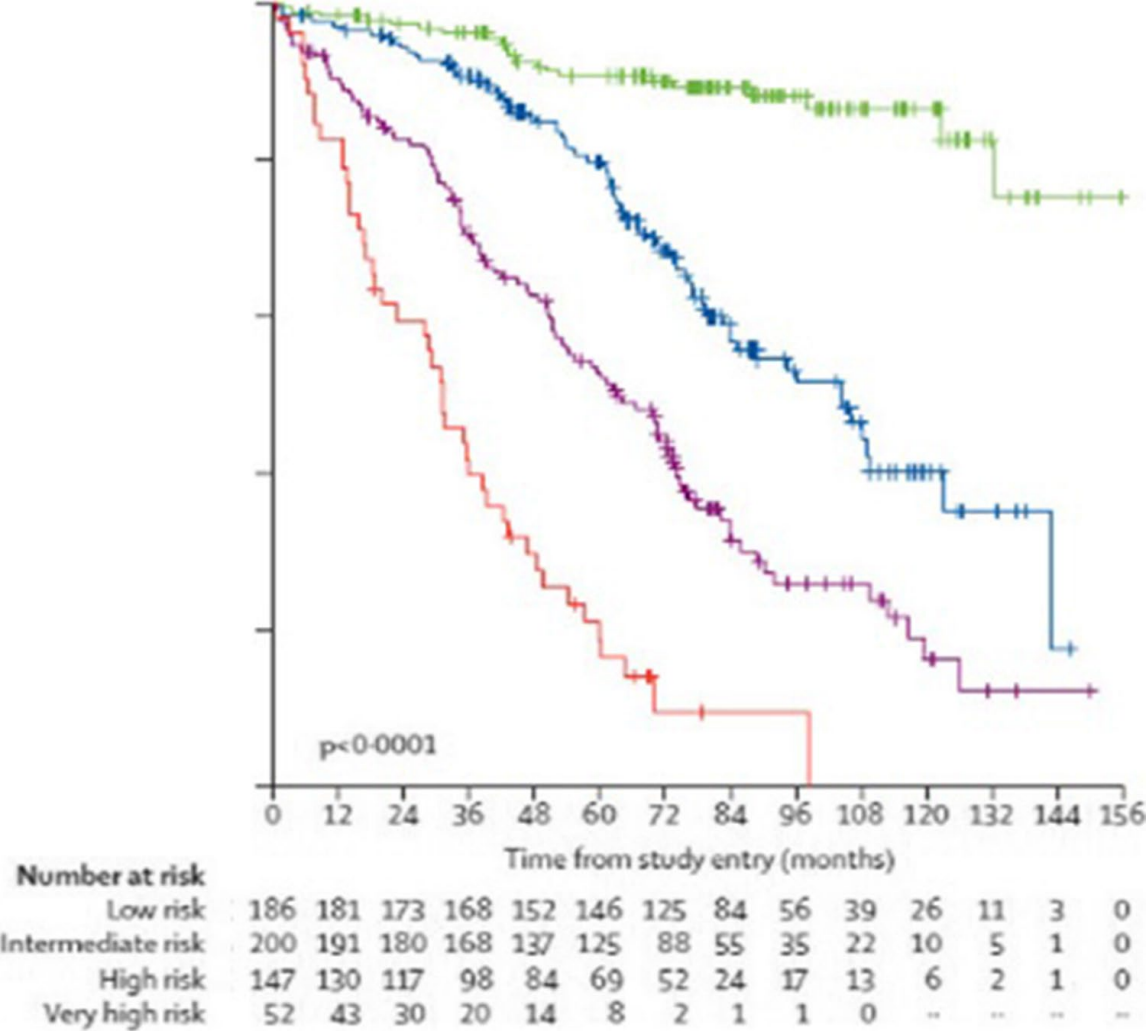
Overall Survival International CLL-IPI Working Group Validation Dataset. Kaplan–Meier overall survival curves for CLL-IPI risk groups. Green = low; Blue = intermediate; Purple = high; Red = very high. Table 2 shows the overall survival statistics. Adapted from [19]

For several decades, simple clinical staging systems (Rai or Binet) have been used to predict prognosis. However, numerous clinical, phenotypic, and molecular genetic features have been associated with outcomes, as noted above. The International Prognostic Index (IPI) for CLL has been developed and validated that considers clinical, laboratory, and genetic features to stratify patients into different risk groups with 5-year overall survival rates ranging from 23 to 93% [19, 31] (Table 2, Fig. 1). Whether this system is applicable in the era of novel agents requires additional study [31].

### Transformation (Richter transformation)

CLL/SLL transformation into an aggressive neoplasm is uncommon (2–10% of cases) and may adopt several forms [32]. In peripheral blood, increased numbers of prolymphocytes, over 55%, are best-termed prolymphocytic transformation of CLL and must be distinguished from the rare de novo B-PLL. Richter transformation (RT) is the term used for CLL/SLL transformation into diffuse large B-cell lymphoma (DLBCL) or rare cases of Hodgkin-lymphoma (HL) [32, 33]. Rarer transformations to plasmablastic lymphoma or transdifferentiation to histiocytic sarcoma have also been reported, particularly under BTK inhibitor therapy [34].

Richter transformation patients are elderly (median age 69 years) and present with elevated lactate dehydrogenase. The median time from diagnosis of CLL/SLL to transformation ranges from 1.8 to 4.7 years for DLBCL-RT and 4.6 to 7.5 years for HL-RT [35]. The rate of transformation has been estimated at approximately 0.5%/year in untreated patients and 1%/year for treated patients. Risk factors for RT include prior therapy [36], BCR stereotype #8, unmutated IGHV, *NOTCH1* mutations, complex karyotypes, high-risk FISH abnormalities (del(17)p, del(11q22)), and CD38 expression [36, 37]. *MYC* rearranged CLL/SLL, a rare occurrence (0.2%), has been associated with prolymphocytic transformation [38]. Most estimates of RT have occurred in the immunochemotherapy-based treatment era, and more recent studies in the era of novel agents suggest the rate has not decreased but further study is needed [39].

**Fig. 2:**
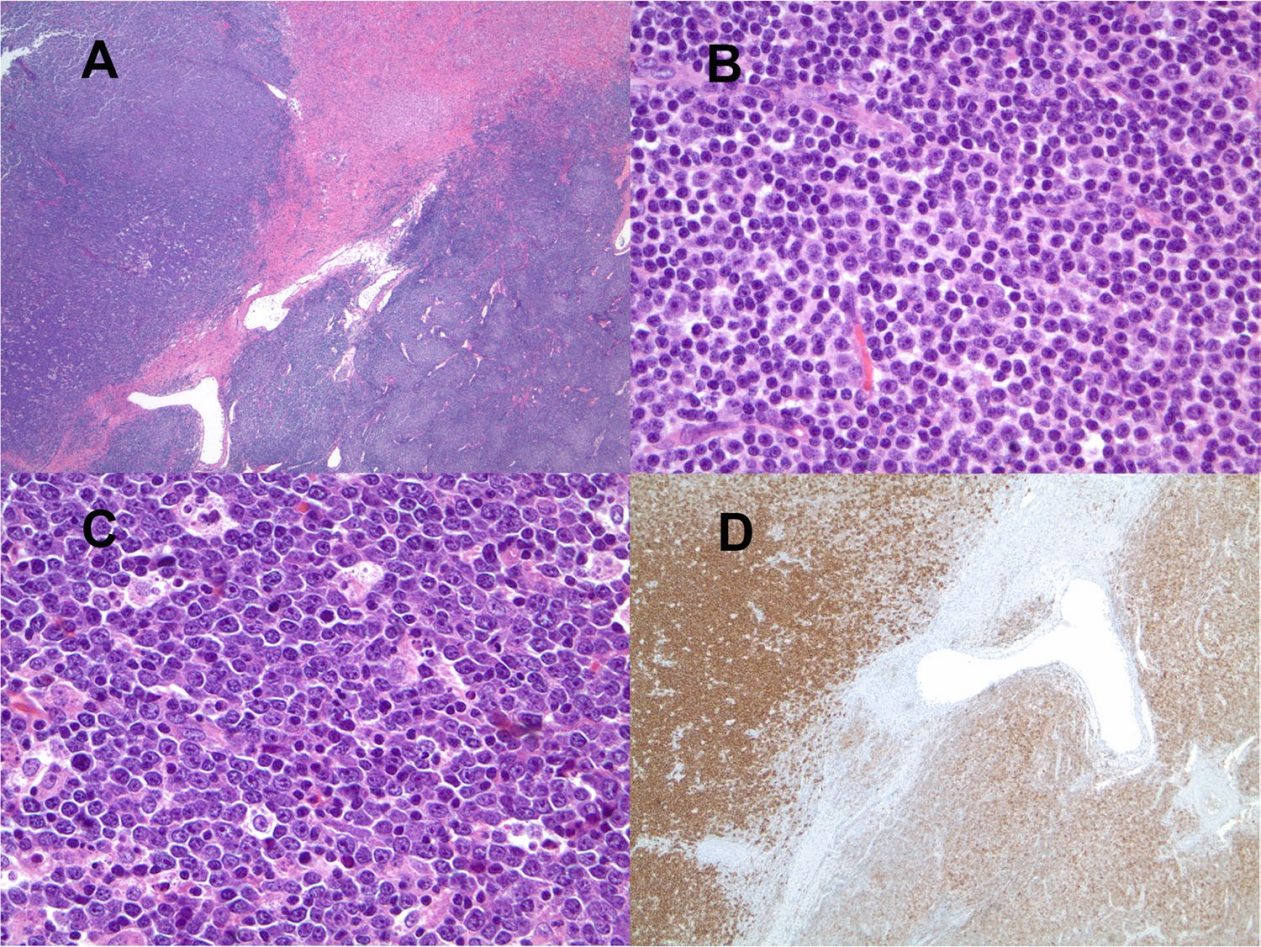
Richter transformation, diffuse large B-cell type. Lymph node biopsy showing composite CLL/SLL and diffuse large B-cell lymphoma. **A** CLL/SLL component in the lower right and Richter transformation in the upper left, hematoxylin, and eosin (H&E, 4 ×). **B** CLL/SLL component with a proliferation centre (H&E, 20 ×). **C** Large cell component with starry sky pattern (H&E, 20 ×). **D** CD20 stain shows weak expression in the CLL/SLL component and stronger CD20 expression in the DLBCL component (CD20 immunohistochemistry, 4 ×). While the CLL/SLL was CD5 + /CD10 − , the large cell component was CD5 − /CD10 + and lacked *MYC* rearrangement by fluorescence in situ hybridization (not shown)

DLBCL is by far the most common histology. Sheets of large, transformed cells are present and should not be confused with expanded proliferation centres containing increased numbers of intermediate-sized prolymphocytes **(**Fig. 2). Most (80%), but not all, cases are clonally related to the CLL/SLL. The proliferative index is high (median 80% Ki67 index) [35].

**Fig. 3:**
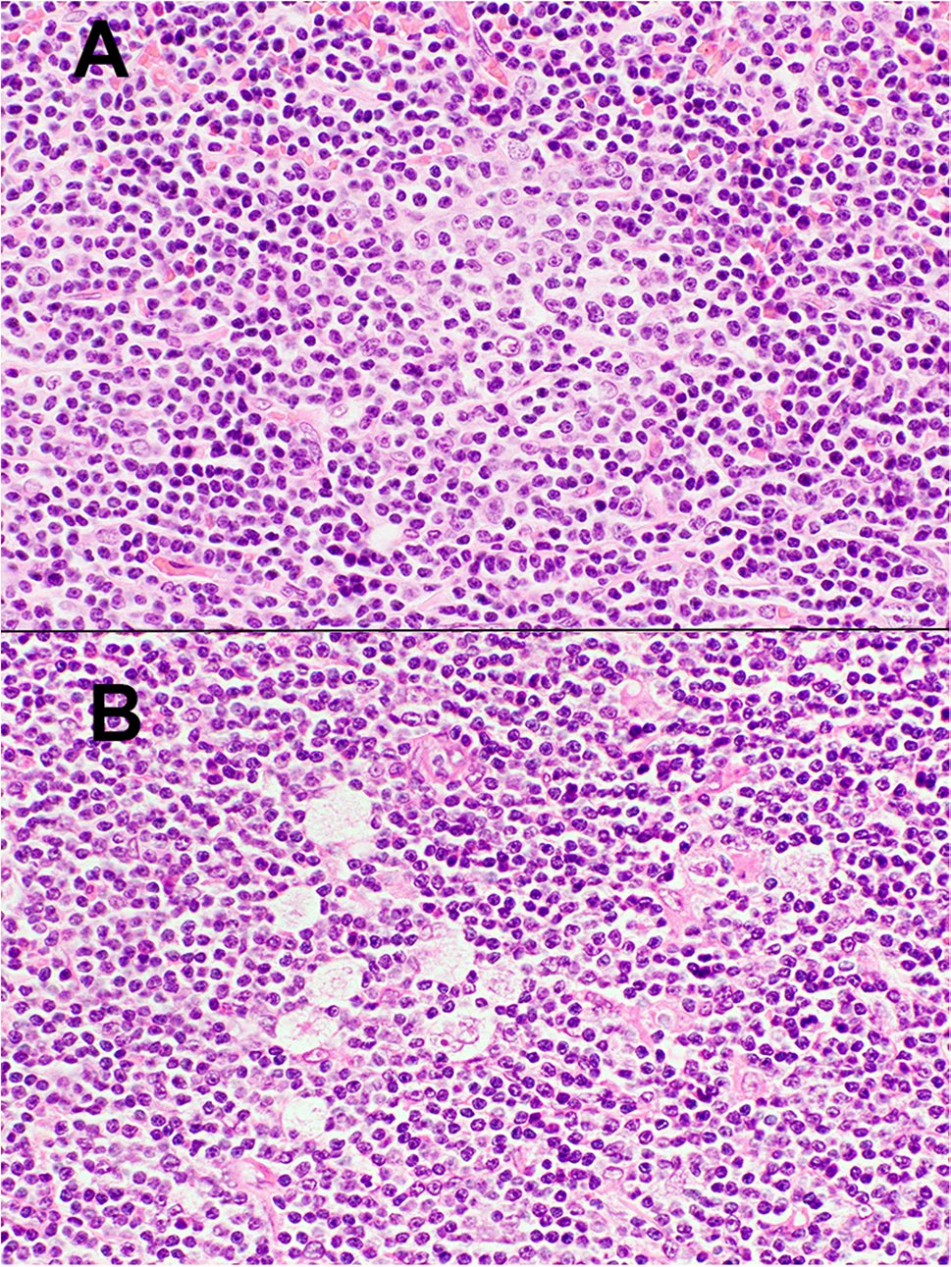
Hodgkin-like Richter transformation, type 1. The upper panel shows a CLL/SLL area containing a proliferation center (H&E, 40 ×). In several areas of the biopsy, admixed Reed-Sternberg cells were present (H&E, 40 ×)

Two types of HL-like Richter transformation have been described. Type 1 shows scattered HRS cells among typical CLL/SLL cells without inflammatory background (Fig. 3). Type 2 resembles classic (c) HL with typical Hodgkin and Reed-Sternberg (HRS) cells in a mixed inflammatory cell infiltrate. Rare cases may show a spectrum of these changes in the same biopsy or in successive samples. The immunophenotype of the HRS cells is similar to cHL with expression of CD15, CD30, and PAX5 but absent CD45. CD20 expression may be seen more frequently in type 2 HRS cells. Clonal relatedness of the HRS cells to the CLL cells has been demonstrated in 29% and 53%, and EBV in 65% and 75% of type 1 and type 2 cases, respectively. There was no relationship between EBV-positivity and clonal relatedness [33].

**Fig. 4:**
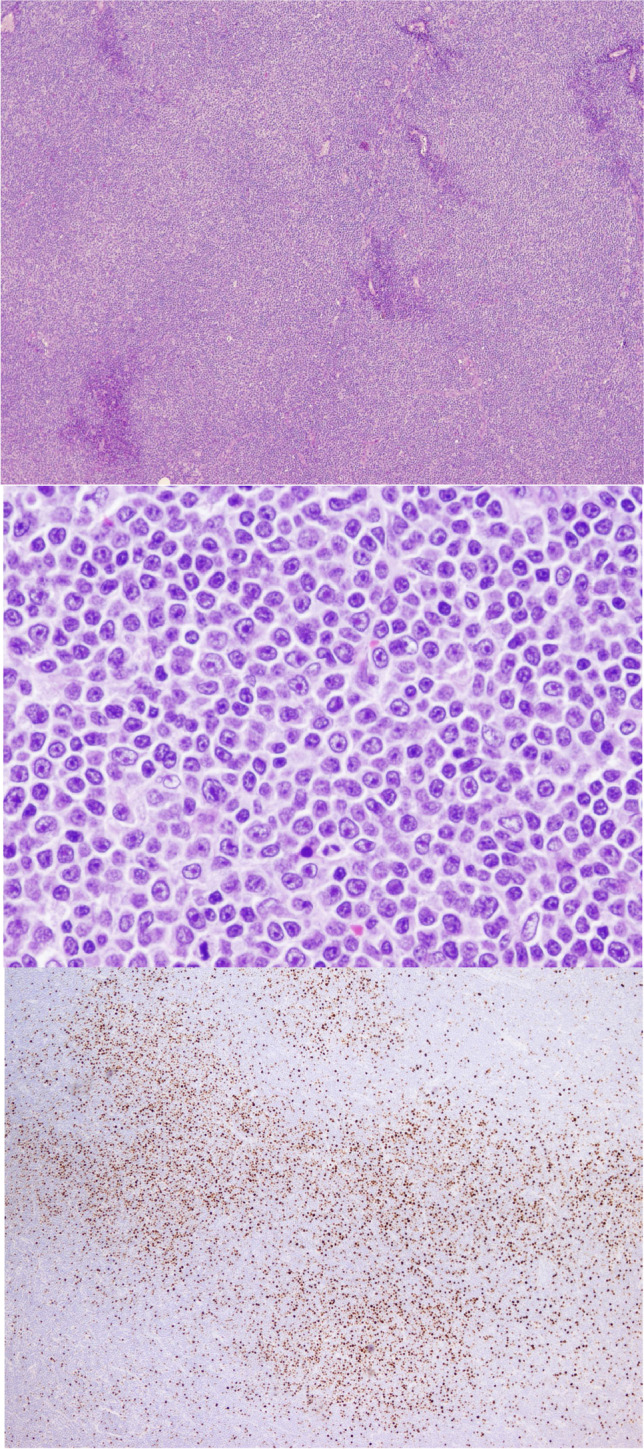
Accelerated CLL/SLL. Lymph node from a patient with CLL/SLL. The upper panel shows that proliferation centres are expanded and confluent (H&E, 4 ×). The middle panel shows that the proliferation centres are composed of larger tumour cells with broader clear cytoplasm and nuclei with prominent nucleoli corresponding to prolymphocytes and paraimmunoblasts (H&E, 40 ×). In the lower panel Ki67 staining shows a high number of positive cells (4 ×)

**Fig. 5:**
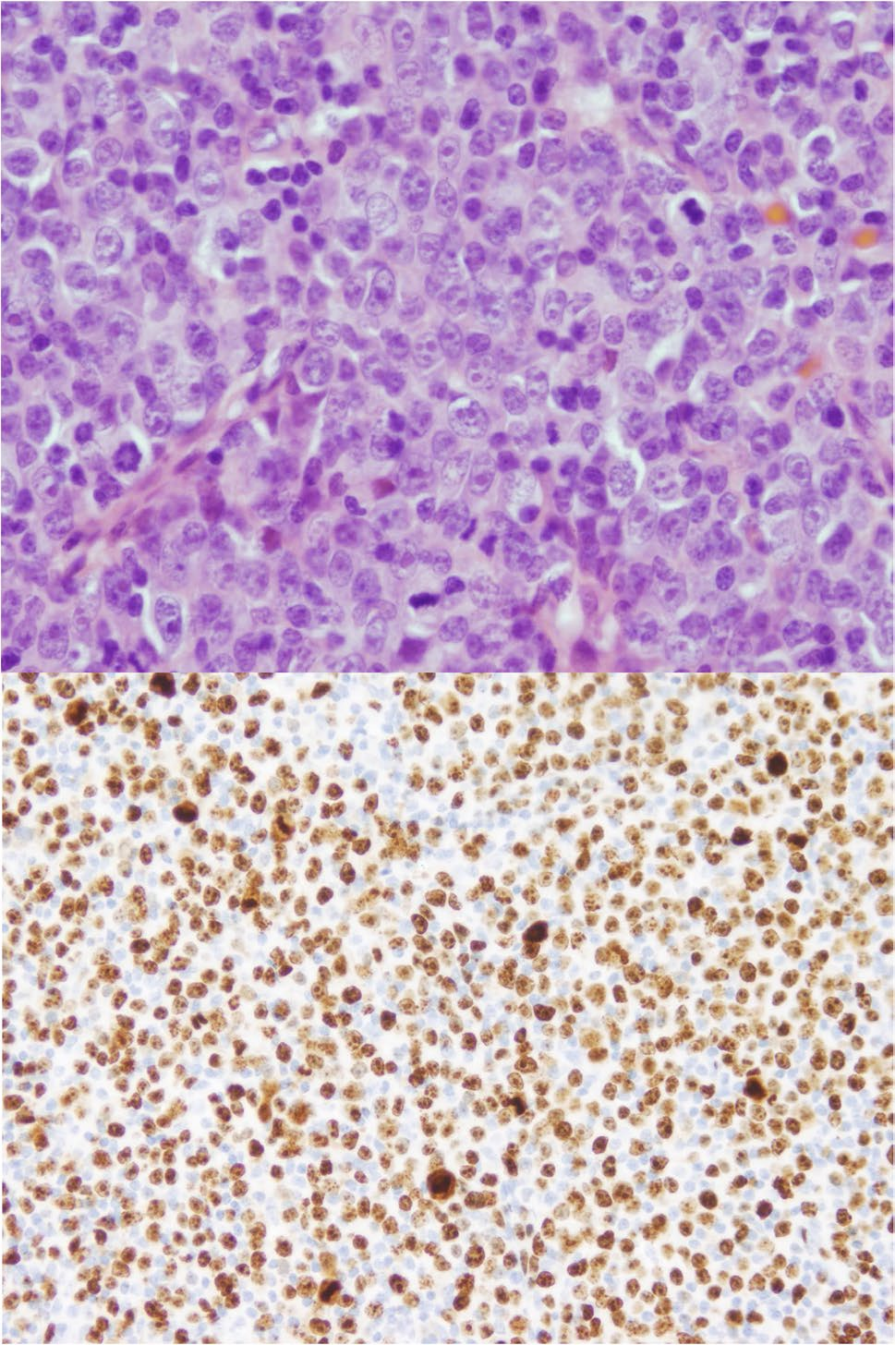
Pseudo-Richter transformation. Tonsil from a patient with CLL. The patient was under treatment with ibrutinib and it was stopped before a surgical procedure. The tonsil grew rapidly and was biopsied. The histological section shows in the upper panel a diffuse proliferation of large cells (H&E, 40 ×). The lower panel shows high proliferation (Ki67, 20 ×). Ibrutinib was reintroduced and the patient is well 7 months later with regression of the disease

Mimics of RT should be recognized. One pitfall is the presence of expanded or confluent proliferation centres such as in accelerated CLL/SLL (Fig. 4). Paraimmunoblasts and prolymphocytes are typically present in proliferation centres. The former is intermediate-sized cells with central nucleoli, open chromatin, and lightly basophilic cytoplasms, and should not be confused with large centroblastic or immunoblastic cells of RT-DLBCL. The phenomenon of “pseudo-Richter transformation” has been reported in patients treated with a BTK inhibitor (Fig. 5). Temporary cessation of ibrutinib in some patients has resulted in clinical and biopsy features of RT that regressed with re-institution of therapy [40].

#### Molecular genetics

DLBCL-type RT appears to lack some of the genetic alterations seen in DLBCL, NOS such as inactivation of *CREBBP/EP300*, *B2M*, and translocations of *BCL2 *or *BCL6*. Recent genomic studies have shown that RT DLBCL integrates alterations in cell cycle regulators (90% of cases) (*TP53*, *CDKN2A/B*, *CDKN1B*), chromatin modifiers (79%) (*SETD2*, *ARIDA/B*), *MYC *(74%), *NF-κB* (74%) (*BIRC3*, *TNFAIP3*, *NFKBIE*), and *NOTCH* (32%) (*NOTCH1*, *SPEN*) pathways with most of these aberrations simultaneously present in most cases with the exception of *MYC* and *NOTCH* alterations that tend to occur in different tumours [36, 41, 42]. Intriguingly, single-cell analysis of sequential samples has identified subclones carrying genetic and transcriptome profiles of the RT already at CLL diagnosis 6–19 years before transformation [41]. RT cells have a high oxidative phosphorylation metabolism that may offer new therapeutic possibilities [41].

#### Prognosis

The prognosis for RT DLBCL type is poor and treatment consists of immunochemotherapy for fit patients. An RT prognostic score based on 5 adverse risk factors (Zubrod performance status > 1, elevated LDH level, platelet count < 100 × 10^9^/L, tumour size ≥ 5 cm, and > 2 prior lines of therapy) has been developed. It stratifies four groups based on the number of risk factors: 0 or 1 = low risk (median survival, 13–45 months); 2 = low-intermediate risk (median survival, 11–32 months); 3 = high-intermediate risk (median survival, 4 months); ≥ 4 = high risk (median survival, 1–4 months) [36, 43]. Clonal relatedness of the DLBCL to the underlying CLL has been reported to predict outcome. Clonally unrelated cases have a much longer survival (approximately 5 years) compared to clonally related DLBCL (8–16 months). Addition of stem cell transplant may be considered for clonally related cases [36].

In HL transformation, the prognosis is poor compared to de novo cHL and is similar in types 1 and 2. HL-directed regimens have been shown to be associated with a favourable outcome compared to CLL-directed therapy in retrospective studies [34, 44, 45].

## B-cell prolymphocytic leukaemia (B-PLL)

**Fig. 6:**
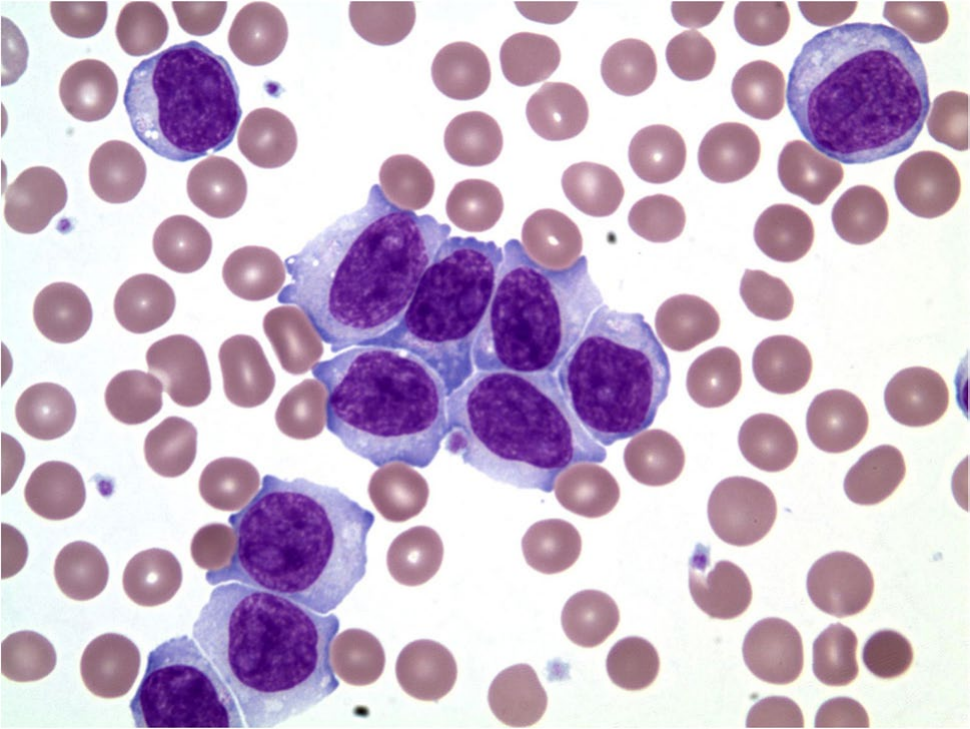
B-PLL. An 84-year-old man presented with marked leukocytosis and anemia. Splenomegaly and mesenteric and retroperitoneal lymphadenopathy was found. The white cell differential showed 82% prolymphocytes. Flow cytometry showed a kappa-restricted B-cell population expressing CD19 and bright CD20 lacking CD5, CD10, CD23, and FMC7. FISH for cyclin D1 rearrangement was negative and karyotyping showed no structural evidence of *CCND1*, *CCND2*, or *CCND3* rearrangement. The blood smear showed numerous prolymphocytes compatible with B-PLL (100 ×). In current practice, a case with this type of morphology would require FISH studies to exclude *CCND1*, *CCND2*, and *CCND3* rearrangements

B-cell prolymphocytic leukemia (B-PLL) is a rare leukaemia characterized by > 55% prolymphocytes in the peripheral blood (Fig. 6). Patients are generally elderly, with B-symptoms, leukocytosis, and marked splenomegaly without lymphadenopathy. The cells are larger than lymphocytes of CLL and have basophilic cytoplasm, open chromatin, and a prominent central nucleolus [46]. The cells express CD19, CD20, CD22, and CD79a, with intense surface IgM/IgD. CD5 and CD23 are reported in 20 and 10% of the cases, respectively [12, 47]. The BCR frequently uses IGHV4-34 and IGHV3-23 and lacks IGHV1-02 usage, the latter found commonly in splenic marginal zone lymphoma [47, 48]. Cases are cytogenetically complex and common chromosomal aberrations include del(17p) (38%), trisomy 18 (30%), del(13q) (29%), trisomy 3 (24%), trisomy 12 (24%), and del(8p) (23%). Recurrent mutations in *TP53*, *MYD88*, *BCOR*, *MYC*, *SF3B1*, *SETD2*, *CHD2*, *CXCR4*, and *BCLAF1* are found, with a subset of cases showing *MYC* translocation [49]*.*

Many cases previously considered as B-PLL harbour the t(11;14)(q13;q32), and are now diagnosed as leukemic variants of blastoid MCL [50]. Some cases of t(11;14) negative PLL have been reported to overexpress *CCND2* or *CCND3*, with variable SOX11 expression suggesting these may also be MCL [51, 52]. However, the expression of *CCND2* or *CCND3* without the presence of a detectable translocation, particularly in SOX11-negative tumours, should not be considered as evidence of MCL since B cell neoplasms express variable levels of *CCND2* or *CCND3* without translocations of these genes [53]. In addition to CLL, transformation to a neoplasm with prolymphocytic features has been described in rare cases of splenic marginal zone lymphomas [54]. These observations and the presence of *MYC* and *TP53* abnormalities in prolymphocytic transformation of CLL raise questions as to whether B-PLL is a discrete entity. However, the differential phenotype and IGHV usage pattern of B-PLL compared to CLL or SMZL and the recognition of B-PLL cases with no evidence of previous small B-cell neoplasm and well-documented absence of *CCND1/D2* or* D3* overexpression suggest that a B-cell neoplasm with B-PLL features distinct from MCL may exist [47, 51, 55]. For these reasons, B-PLL is retained in the 2022 ICC for de novo cases with demonstrated absence of *CCND1/2/3* translocation [2]. This recognition will help to better delineate this category in future studies.

## Mantle cell lymphoma

Mantle cell lymphoma (MCL) constitutes 5–7% of all lymphomas in the Western world and occurs more frequently in males (M:F = 3:1) with median age at diagnosis of approximately 65 years. MCL is clinically heterogenous with a subgroup of patients that do not require treatment for several years to highly aggressive MCL with dismal prognosis [56, 57]. Two subtypes of MCL exist; conventional MCL (cMCL) and non-nodal leukemic MCL (nnMCL) (reviewed in [1]). The present review focuses on new aspects of this disease including an update from the 2022 ICC of lymphoid malignancies [2].

### Early lesions of MCL

Sensitive methods can detect rare cells carrying t(11;14) in blood of healthy individuals, occurring in < 10% of individuals tested (reviewed in [1]). A precursor lesion of MCL, in situ mantle cell neoplasia, can incidentally be found in lymph nodes [58, 59]. In situ mantle cell neoplasia is characterized by few cyclin D1 positive cells carrying the t(11;14) translocation in a thin rim around reactive germinal centres without expansion of the mantle zone. The cells can sometimes lack CD5 expression and SOX11 can be positive or negative. This precursor lesion should be distinguished from MCL with a mantle zone pattern since it rarely progresses to overt MCL. This situation should also be distinguished from some CLL/SLL cases in which the tumour cells have a perifollicular involvement that, in contrast to in situ mantle cell neoplasia, tend to involve the outer region of the mantle [60]. These morphological observations suggest that similar to MCL, the normal cell counterpart of CLL/SLL may be also related to mantle cells, at least in a subset of cases.

### Cytomorphology, growth pattern, and phenotype

**Fig. 7:**
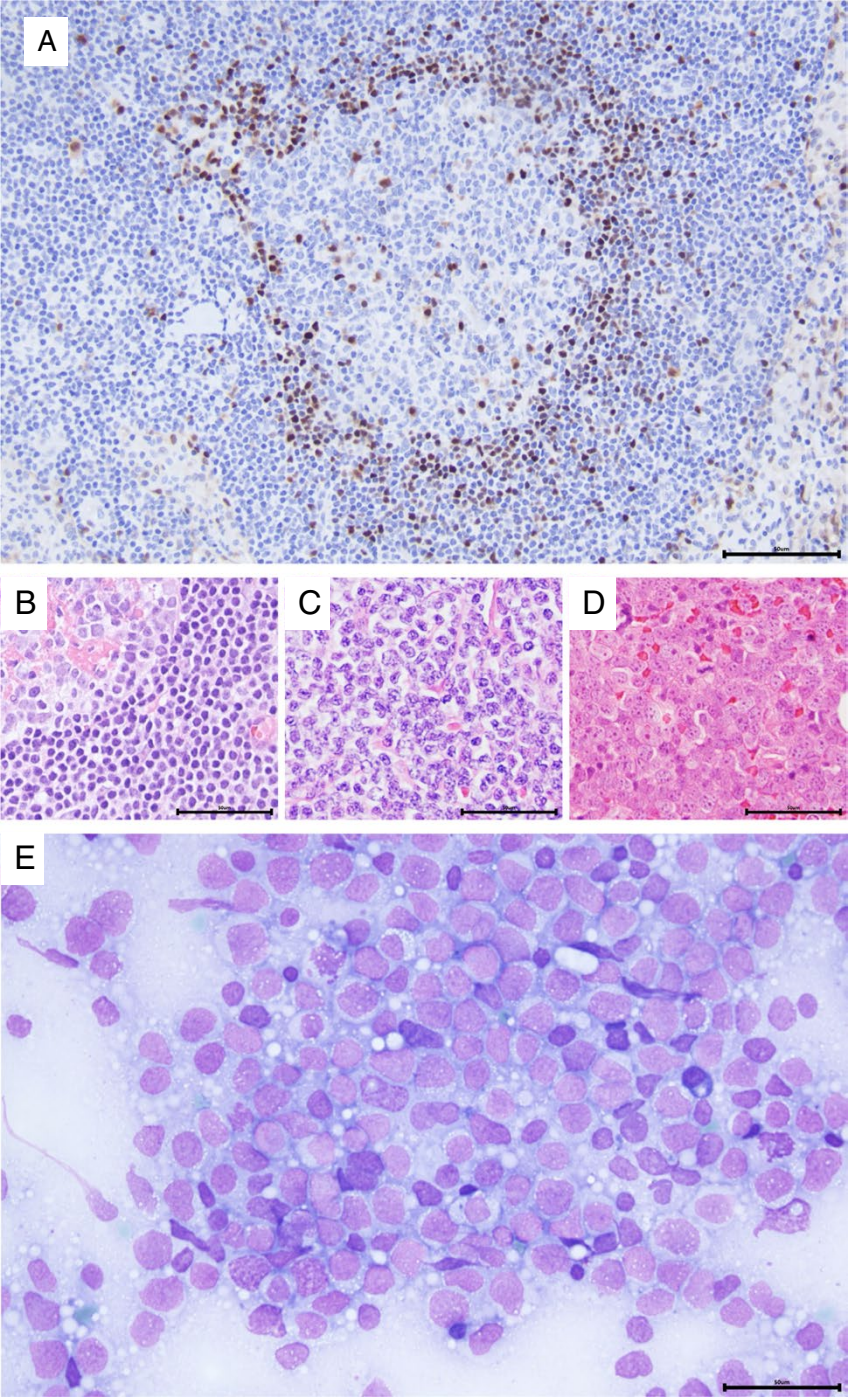
Cytomorphological variants of MCL. **A** MCL with mantle zone growth pattern as illustrated with cyclin D1 staining. **B** H&E staining of the same case showed small cell cytomorphology, part of a residual germinal centre is seen in the upper left corner. **C** Centrocytic variant. The nuclei are irregular and cleaved. **D** Blastoid variant in H&E staining. Cells are medium-sized to large with visible nuclei. **E** Imprints of the same case show medium-sized to large cells with immature chromatin and vacuolated cytoplasm. The scale bar is 50 μM

**Fig. 8:**
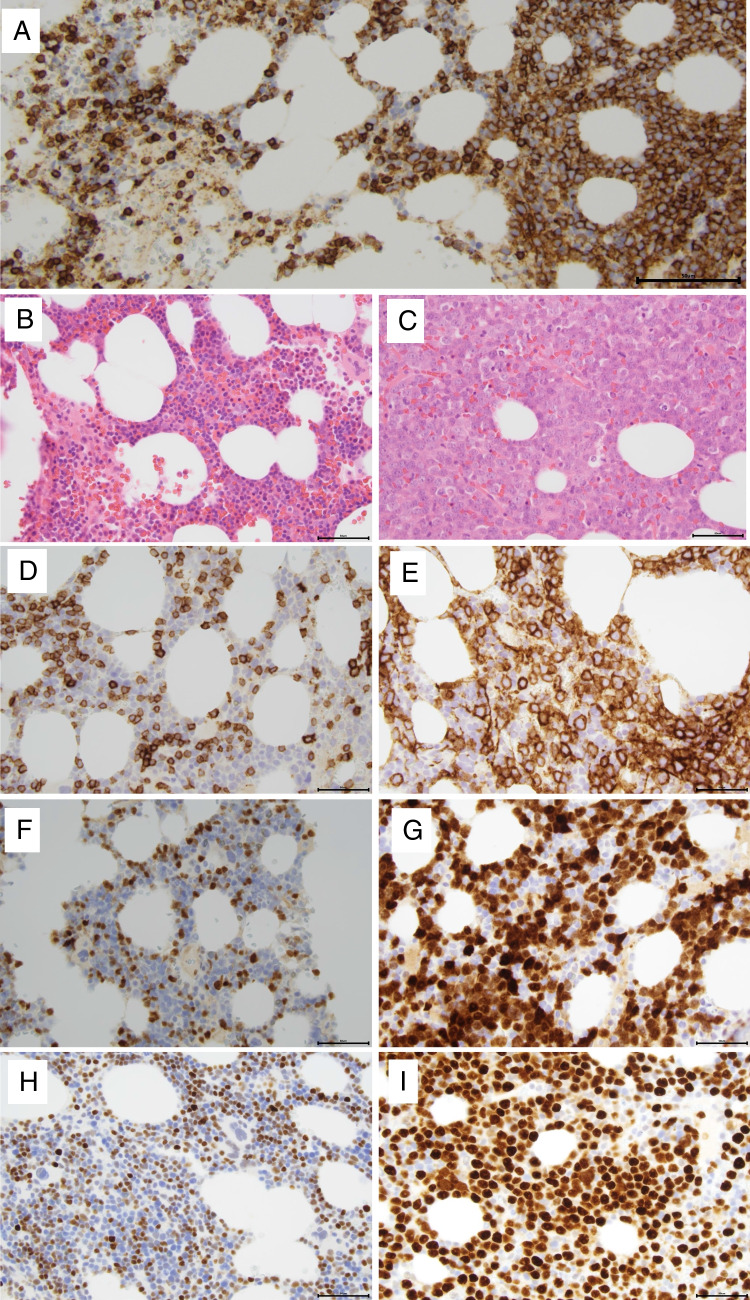
MCL, SOX11 negative, with a small cell and a blastoid component in the bone marrow. Bone marrow biopsy from a patient with lymphocytosis. In the blood, the cells were positive for CD19, CD20, CD5, CD79b, and kappa, while CD43 and CD10 were negative, and CD23 and CD200 were dim. The bone marrow flow cytometry showed 2 kappa positive cell populations, one small cell population CD20 + + , CD5 + , CD23 + , CD79b + , CD200 partial, CD10 − , and one large cell population CD20dim, CD10 + , CD79b + , and CD5 − , CD23 − and CD200 − . **A** CD20 staining with the small cell population to the left and the blastoid component to the right. **B** The small cell population distributes in a lace-like pattern between myelopoiesis and fat cells. **C** The blastoid component grows in dense sheets. **D** CD5 is expressed in the small cells with a weaker intensity that in T-cells. SOX11 was negative (not shown) (**E**). CD10 is expressed in the blastoid component. The blastoid cells expressed MUM1 and variable BCL6 and were negative for SOX11 (not shown) (**F**–**G**) Cyclin D1 + in both components. (H-I) p53 + in both components. *TP53* sequencing showed pathogenic *TP53* mutation in codon 258. FISH for *CCND1* break-apart was positive in both components while FISH for *MYC*, *BCL2*, and *BCL6* were negative. The scale bar is 50 μM

Based on cytology, four major variants are recognized: small cell, classic (centrocytic), pleomorphic with cells larger than the classic variant, and a blastic variant with cells resembling lymphoblasts, the latter two are sometimes grouped as blastoid MCL [57] (Fig. 7). Rarely, MCL can have a cell morphology reminiscent of marginal zone cells with clear cytoplasm [57]. The cytology might change at relapse, often to the more aggressive blastoid variant but also the reverse can occur [61]. Three growth patterns are recognized: mantle zone with thickened mantle zones surrounding preserved non-malignant germinal centres, nodular, and diffuse. Most cases of MCL have the following phenotype: CD5 + , CD19 + , CD20 + , surface IgM + /IgD + , Cyclin D1 + , SOX11 + , BCL2 + , BCL6 − , CD10 − , CD23 − , CD200 − , but aberrant phenotypes are seen in 10–15% of cases which might cause diagnostic difficulties. Lack of CD5 is seen in approximately 5–10% of cases and has been associated with better patient outcomes independent of favourable prognostic markers such as nnMCL, SOX11 negativity and low tumour cell proliferation [62]. Positivity for CD10 and/or BCL6 has been reported in approximately 16% of cyclin D1 + , SOX11 + MCL and is associated with higher proliferation, blastoid morphology, and 3q27 amplifications, while translocations involving *BCL6* are very rare in MCL [63] (Fig. 8).

### Molecular pathogenesis of MCL

The genetic hallmark of MCL is *CCND1* rearrangement that juxtaposes the cyclin D1 gene, *CCND1*, to the immunoglobulin heavy or light chain loci [57]. However, a few cases with morphology, phenotype, gene expression profile, and clinical behaviour typical for MCL lack cyclin D1 expression and t(11;14). In most of these cyclin D1-negative MCLs, translocations of *CCND2* or *CCND3* can be demonstrated by FISH [52]. Recent studies have shown that these translocations may be cryptic, with only the immunoglobulin enhancer juxtaposed to *CCND2* or *CCND3*, upregulating their expression. Importantly, immunohistochemical staining for cyclin D2 or cyclin D3 is not specific but these cases can be identified by their high expression of *CCND2* or *CCND3* mRNA [57].

The t(11;14) translocation is an early event in MCL pathogenesis and occurs in precursor B-cells in the bone marrow and only rarely in mature B-cells [64]. Dysregulation of cyclin D1 impairs cell cycle regulation but does not alone lead to lymphoma development [65]. MCL is in general a genetically unstable lymphoma and the presence of a complex karyotype is associated with an unfavourable prognosis [66]. Secondary genetic events (gene mutations and genetic lesions) in MCL are associated with genetic instability (*ATM*, 40–50% of cases, *TP53*, 15–20%), lead to cell cycle dysregulation (*CDKN2A*, 20–25%, *CCND1*, 20%, and *MYC*), induce changes in immune response and B-cell receptor signalling (*CARD11*,* BTK*, *TLR2*, *S1PR1*, and others), affect epigenetic and chromatin modifiers (*SMARCA4*, *ARID1B*, *TET2*, *KMT2D*, and others), dysregulate *NOTCH* signalling, and involve genes coding for protein ligases such as *UBR5* [64]. Some studies have analysed gene mutations at diagnosis and after relapse post-chemoimmunotherapy, showing an increased mutation frequency at disease progression [67–69].

### MCL with indolent clinical behaviour

**Fig. 9:**
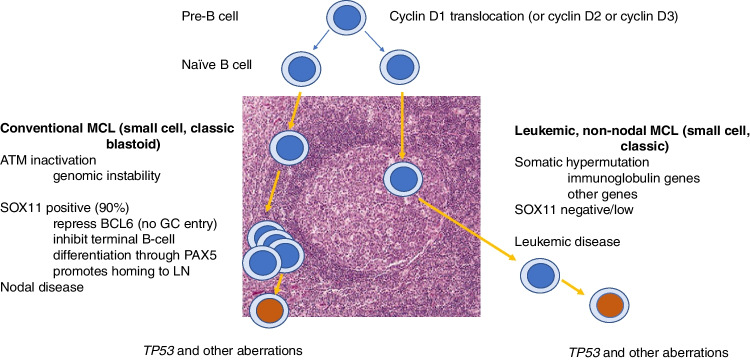
Conventional and non-nodal, leukemic MCL. Both MCL variants are derived from a precursor cell that acquired a cyclin D1 (or cyclin D2 or cyclin D3) translocation at the pre-B cell stage. Conventional MCL (to the left) is hypothesised to follow a differentiation pathway similar to naïve pre-germinal B cells and most cases have unmutated IGHV genes. *ATM* inactivation is a common feature and is associated with genomic instability. Most conventional MCL express SOX11, a transcriptional regulator which has been shown to impair terminal B cell differentiation in MCL. Non-nodal, leukemic MCL (to the right) is thought to originate from the antigen-experienced post-germinal centre/memory B cells and shows evidence of somatic hypermutation in IGHV and other genes such as *CCND1*. SOX11 is generally not expressed in non-nodal, leukemic MCL. Both MCL variants can acquire *TP53* mutations (indicated in red) which predict an aggressive disease course and inferior outcome in MCL

In spite of novel treatment strategies, MCL is still considered an incurable lymphoma. However, there are cases with a more indolent clinical behaviour, of which some belong to the recently recognized nnMCL variant [1, 46]. These patients have minimal lymphadenopathy and present with lymphocytosis, bone marrow involvement, and splenomegaly. The actual frequency of nnMCL is estimated to be 10% of MCL, but since these cases may have a similar clinical presentation as CLL, behave indolently and do not need immediate treatment at diagnosis they might be incompletely worked up at diagnosis which limits a precise estimation of the incidence. Although the t(11;14) breakpoints and the underlying mechanisms for translocation are similar in nnMCL and cMCL [64] there are several morphological and genetic features that distinguish nnMCL and cMCL. By flow cytometry, the phenotype of nnMCL might be aberrant with negativity for CD5, and positivity for CD23 or CD200 [70, 71]. Some cases may have plasmacytic differentiation that is not seen in conventional MCL [72]. Typically, nnMCL lack or have very low expression of the transcriptional regulator SOX11. In the bone marrow, the growth pattern might be lace-like with scattered cells infiltrating in between myelopoiesis and fat cells (Fig. 8) [70]. The cells are often small, and cell proliferation is low (< 10%). In contrast to most cMCL, nnMCL has hypermutated IGHV genes and is thought to correspond to memory B-cells that have passed the germinal centre (Fig. 9). Whole genome sequencing of 21 nnMCL and 60 cMCL showed that nnMCL had less genomic complexity, lacked *ATM* mutations, had fewer epigenetic mutations, and had more frequent mutations of *CCND1* and *TLR2*. Losses of chromosome 17p and *TP53* mutations were slightly enriched in nnMCL (Table 3) [64].

**Table 3:** Features of conventional MCL (cMCL) and leukemic non-nodal MCL (nnMCL). Data from [64]

Feature	cMCL	nnMCL
Translocations	*CCND1*, *CCND2* or* CCND3*	*CCND1*, *CCND2* or *CCND3*
Lymphadenopathy	Yes	No/minimal
SOX11 +	Yes	No/minimal
IGHV mutations	No/limited	Yes
Genomic complexity	High	Low
*ATM* aberration	Frequent (40–50%)	No
*CCND1* mutation	No	Yes
*TP53* aberrations	15–20%	20–30%

Some cMCL patients qualify for watchful waiting and are clinically characterized by limited disease, lack of large lymph nodes and spleen, and a low Mantle cell lymphoma International Prognostic Index score (MIPI: based on age, performance status, lactate dehydrogenase and leukocyte count [73]). Patients with isolated involvement of the gastrointestinal tract (lymphomatoid polyposis) may have a very indolent disease course [74, 75]. Indolent cases of cMCL are of small or classic morphology (not blastoid/pleomorphic), with low tumour cell proliferation (< 10% Ki-67) and lack of strong p53 expression [56, 76–79].

### Prognostic factors

High tumour cell proliferation (> 30% Ki-67) and blastic/pleomorphic morphology reflect a high genetic complexity and are unfavourable prognostic features that should always be described in the pathology report [2]. A 35 gene proliferation signature (MCL35) applicable in formalin-fixed tissue is strongly associated with prognosis [80, 81]. Also, *TP53*, *NOTCH1* and *CDKN2A* aberrations are associated with dismal prognosis in patients receiving intensive immunochemotherapy [78]. In general, there is a good agreement between immunohistochemistry (IHC) for p53 and the presence of *TP53* mutations [82] unless the epitopes recognized by anti-p53 antibodies are lost due to mutations. The 2022 ICC recommends including *TP53* mutation analysis in the diagnostic workup of MCL [2].

Rare cases of MCL have *MYC* amplifications or rearrangement [64, 83, 84]. These cases are often blastic or pleomorphic with high tumour cell proliferation, high genomic complexity, may express TdT, and may lack SOX11 [84]. While such a finding should be reported, these cases should not be called “double-hit” lymphomas, lymphoblastic leukemias/lymphomas, or be included in the “high-grade B-cell” category [2]. It should also be mentioned that other lymphoid malignancies such as CLL/SLL, follicular lymphoma and large cell B-cell lymphomas may acquire *CCND1* aberrations during the disease course (as discussed in [2]). In large B-cell lymphomas, the *CCND1* rearrangement is frequently associated with *MYC*,* BCL2* and *BCL6* translocations. These aggressive lymphomas should not be diagnosed as MCL and genomic studies are valuable in differentiating between the entities [85].

### SOX11 in MCL

SOX11 is a transcriptional regulator that is expressed during embryogenesis but not in normal, mature lymphocytes. SOX11 is aberrantly expressed in MCL and it has a diagnostic value since it identifies cyclin D1 negative cases. SOX11 mRNA is highly variable in MCL with a Gaussian distribution of the mRNA levels that correlates with protein expression, and therefore, a definite cut-off to call a case SOX11 positive is difficult to establish [86, 87]. Still, SOX11 together with other morphological, clinical, and genetic features serves to discriminate the nnMCL. An IHC cut-off of 10% SOX11-expressing cells has been suggested in cMCL based on the different outcomes of cMCL with low/absent SOX11 expression in the setting of clinical trials [77]. A gene signature including SOX11 and 15 other genes distinguishes cMCL from nnMCL [88] but is not widely used.

In MCL SOX11 expression is induced by a distant super enhancer [89]. Several studies have investigated the function of SOX11. SOX11 seems to regulate cell differentiation by enhancing BCR signalling, suppressing *BCL6* expression, and upregulating *PAX5* leading to lack of germinal centre transition and block of terminal B-cell differentiation. SOX11 also affects cell migration and adhesion which could lead to cell-adhesion-mediated drug resistance (reviewed in [90]).

## Summary

CLL/SLL and MCL are two entities sharing an origin in cells that have not experienced the germinal centre and carry unmutated or low load of IGHV mutations, or in memory-like cells that have passed through the germinal centre and carry mutated IGHV. These two different cells of origin determine different biology in their respective derived tumours in CLL/SLL and MCL. Despite similar cells of origin, CLL/SLL and MCL differ in their molecular and genetic pathogenic mechanisms, morphological and phenotypic characteristics, and clinical manifestations. The management of both diseases is rapidly evolving with the incorporation of novel agents that are offering new perspectives. Early lesions are recognized in both entities, and, on the other end of the spectrum, both can progress and transform to very aggressive lymphomas that represent a current challenge for their treatment. The diagnosis of these entities is incorporating new requirements of molecular and genetic information that are progressively modulating therapeutic decisions.
