# Correction to: Chronic lymphocytic leukaemia/small lymphocytic lymphoma and mantle cell lymphoma: from early lesions to transformation

**DOI:** 10.1007/s00428-023-03488-8

**Published:** 2023-01-12

**Authors:** Birgitta Sander, Elias Campo, Eric D. Hsi

**Affiliations:** 1 Department of Laboratory Medicine, Division of Pathology, Karolinska Institutet and Karolinska University Hospital, Stockholm, Sweden; 2 Laboratory of Pathology Hospital Clinic of Barcelona, University of Barcelona, Barcelona, Spain; 3 Institute of Biomedical Research August Pi I Sunyer (IDIBAPS), Barcelona, Spain; 4 Department of Pathology, Wake Forest University School of Medicine, Winston-Salem, NC USA

**Correction to: Virchows Archiv**

**https://doi.org/10.1007/s00428-022-03460-y**

The authors regret that the published version of the above article contained an error.

The types of Hodgkin lymphoma (HL)-like Richter transformation were mislabeled in the manuscript on page 5, paragraph 3. Currently it reads:

“Two types of HL-like Richter transformation have been described. Type 1 resembles classic (c) HL with typical Hodgkin and Reed-Sternberg (HRS) cells in a mixed inflammatory cell infiltrate. Type 2 shows scattered HRS cells among typical CLL/SLL cells without inflammatory background (Fig. 3). Rare cases may show a spectrum of these changes in the same biopsy or in successive samples. The immunophenotype of the HRS cells is similar to cHL with expression of CD15, CD30, and PAX5 but absent CD45. CD20 expression may be seen more frequently in type 2 HRS cells. Clonal relatedness of the HRS cells to the CLL cells has been demonstrated in 29% and 53%, and EBV in 65% and 75% of type 1 and type 2 cases, respectively. There was no relationship between EBV-positivity and clonal relatedness [33].”

This should be corrected to (the altered text is highlighted in bold):

“Two types of HL-like Richter transformation have been described. **Type 1 shows scattered HRS cells among typical CLL/SLL cells without inflammatory background (Fig. 3). Type 2 resembles classic (c) HL with typical Hodgkin and Reed-Sternberg (HRS) cells in a mixed inflammatory cell infiltrate.** Rare cases may show a spectrum of these changes in the same biopsy or in successive samples. The immunophenotype of the HRS cells is similar to cHL with expression of CD15, CD30, and PAX5 but absent CD45. CD20 expression may be seen more frequently in type **1** HRS cells. Clonal relatedness of the HRS cells to the CLL cells has been demonstrated in 29% and 53%, and EBV in 65% and 75% of type 1 and type 2 cases, respectively. There was no relationship between EBV-positivity and clonal relatedness [33].”

In addition, the figure 3 legend should be corrected to read (altered text is highlighted in bold):

Fig. 3** HL-like Richter transformation, type 1**. The upper panel shows a CLL/SLL area containing a proliferation center (H&E, 40 ×). In several areas of the biopsy **(lower panel)**, admixed Reed-Sternberg cells were present (H&E, 40 ×)


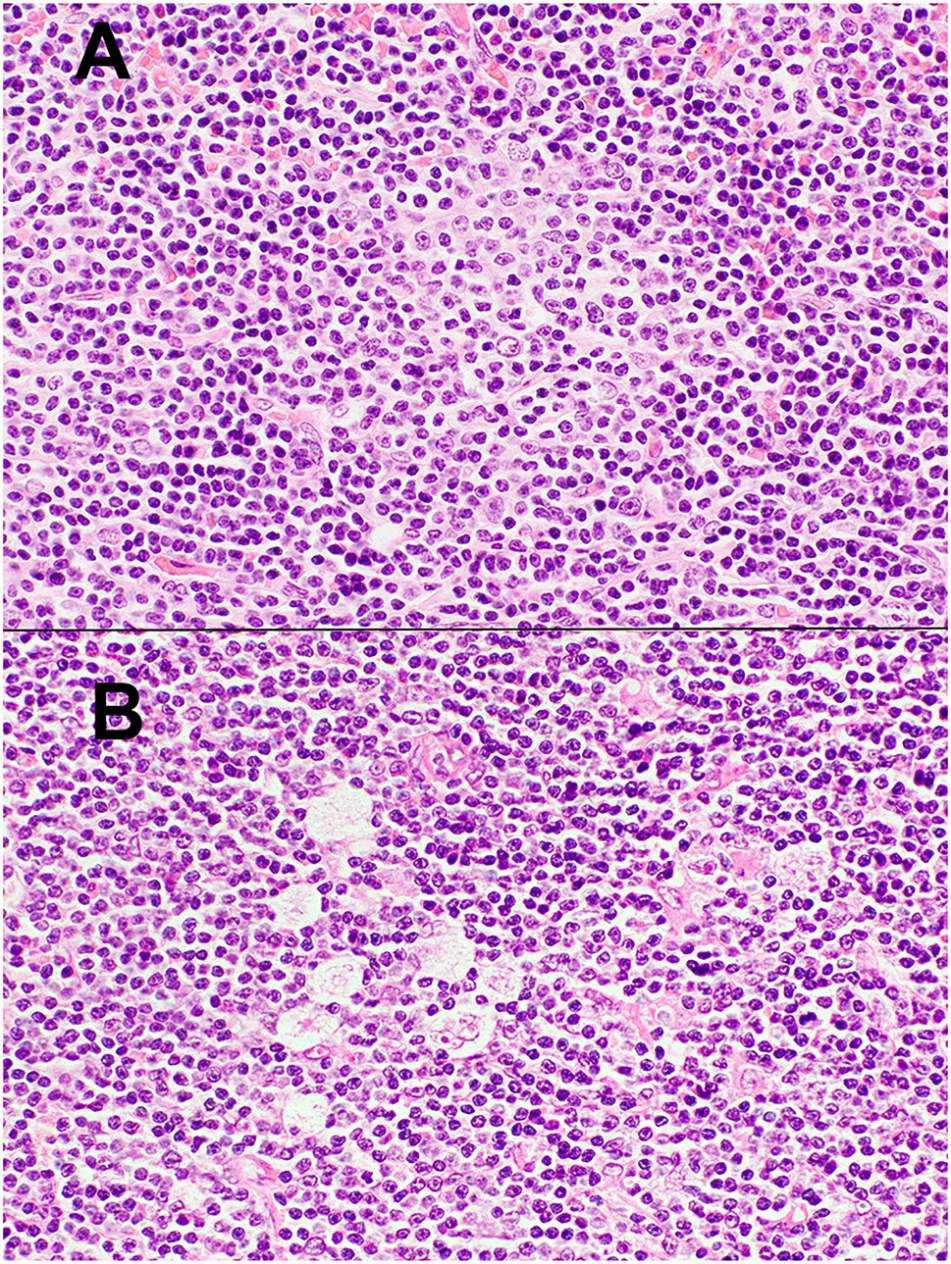


The original article has been corrected.

